# Development of an RT-LAMP Assay for Detecting *tet*(M) in *Enterococcus* Species: Enhancing AMR Surveillance Within the One Health Sectors

**DOI:** 10.3390/diagnostics15101213

**Published:** 2025-05-12

**Authors:** Ebthag A. M. Mussa, Anis Rageh Al-Maleki, Musheer A. Aljaberi, Abdulsamad Alsalahi, Mohd Nasir Mohd Desa, Azmiza Syawani Jasni, Siti Zubaidah Ramanoon, Atiyeh M. Abdallah, Rukman Awang Hamat

**Affiliations:** 1 Department of Medical Microbiology, Faculty of Medicine and Health Sciences, Universiti Putra Malaysia, Serdang 43400, Selangor, Malaysia; ebtihaj.almabrok@gmail.com (E.A.M.M.); azmiza@upm.edu.my (A.S.J.); 2 Department of Medical Microbiology, Faculty of Medicine, Universiti Malaya, Kuala Lumpur 50603, Malaysia; anisrageh@um.edu.my; 3 Department of Internal Medicine, Section Nursing Science, Erasmus University Medical Center (Erasmus MC), 3015 GD Rotterdam, The Netherlands; m.al-jaberi@erasmusmc.nl; 4 Research Centre Innovations in Care, Rotterdam University of Applied Sciences, P.O Box 25035, 3001 HA Rotterdam, The Netherlands; 5 Department of Pharmacology, Faculty of Pharmacy, Sana’a University, Mazbah District, Sana’a Secretariat 1247, Yemen; ahmedsamad28@yahoo.com; 6 Department of Biomedical Sciences, Faculty of Medicine and Health Sciences, Universiti Putra Malaysia, Serdang 43400, Selangor, Malaysia; mnasir@upm.edu.my; 7 Department of Farm and Exotic Animal Medicine and Surgery, Faculty of Veterinary Medicine, Universiti Putra Malaysia, Serdang 43400, Selangor, Malaysia; sramanoon@upm.edu.my; 8 Department of Biomedical Sciences, College of Health Sciences, QU Health, Qatar University, Doha 2713, Qatar

**Keywords:** enterococci, tetracycline resistance, *tet*(M), RT-LAMP, antimicrobial-resistant, One Health

## Abstract

The increasing prevalence of antimicrobial-resistant (AMR) bacteria in humans, animals, and the environment underscores the necessity for a rapid, sensitive, and specific method to identify resistance genes. **Objectives**: This study aims to develop a reliable detection tool for identifying the tetracycline-resistant gene *tet*(M) in *Enterococcus* species using a real-time loop-mediated isothermal amplification (RT-LAMP) assay. Real-time visualization through a turbidimeter enabled precise estimation of time-to-positivity for gene detection. **Methodology**: Six primers were designed using PrimerExplorer v.5, and the assay was optimized across different temperatures and incubation times. Validation was conducted by testing 52 *tet*(M)-positive clinical enterococci isolates and spiking urine samples from a healthy volunteer and a cow with *tet*(M)-positive *Enterococcus* species. **Results:** The *tet*(M) gene was detected as early as 33 min, with optimal amplification occurring within 60 min at 60 °C. The assay demonstrated 100% specificity with the established primers. The sigmoidal graphs were corroborated with visual confirmation methods, including a green color change (visible to the naked eye), green fluorescence (under UV light), and a 200 bp PCR product observed via agarose gel electrophoresis. Notably, the *tet*(M) RT-LAMP assay exhibited a detection limit of 0.001 pg/μL, significantly surpassing conventional PCR, which had a detection limit of 0.1 pg/μL. **Conclusions**: This rapid, cost-effective, highly sensitive, and specific *tet*(M) RT-LAMP assay holds significant promise as a surveillance tool for antimicrobial resistance monitoring within a One Health framework, particularly in low-resource countries.

## 1. Introduction

Enterococci are a group of Gram-positive, non-sporulating, and facultative anaerobic bacteria encompassing 60 species that are prevalent in various sources, including humans, animals, plants, soil, and the environment [1,2]. Enterococci, while commonly regarded as harmless inhabitants of the gastrointestinal tracts in both humans and animals, possess the potential to induce opportunistic infections, leading to a range of human and animal illnesses [1]. Furthermore, the overuse of antibiotics, especially broad-spectrum antibiotics, can lead to the emergence of antimicrobial resistance due to selective pressure [3]. Considering the rise of multidrug-resistant isolates worldwide, it is worth noting that two enterococcal species, namely *Enterococcus faecalis* and *Enterococcus faecium*, have emerged as significant contributors to human infections in hospital environments [1]. These two enterococcal species have become the second most common pathogens associated with healthcare-related infections, particularly catheter-associated urinary tract infections (CAUTIs) and central-line-associated bloodstream infections [4]. Extensive research has documented the clinical challenges associated with managing enterococcal infections, primarily due to the increasing resistance to multiple antibiotic classes, including β-lactams, macrolides, fluoroquinolones, glycopeptides, and tetracyclines [1,4,5,6,7,8]. Tetracyclines are broad-spectrum antimicrobial agents against various types of Gram-positive and Gram-negative bacteria. They are commonly used for systemic and localized infections to inhibit bacterial growth [9,10]. However, the emergence of novel tetracycline derivatives, including eravacycline, omadacycline, and tigecycline, has raised significant concerns regarding the inappropriate use of these antibiotics, particularly in relation to the development of tetracycline-resistant bacteria, notably enterococci [11,12,13]. This issue is exacerbated by the significant increase in tetracycline-resistant enterococci observed across clinical, animal, and environmental isolates [9,10,14].

The World Health Organization (WHO) has recognized loop-mediated isothermal amplification (LAMP) as a valuable tool in antimicrobial resistance (AMR) surveillance. The WHO 2014 global report on AMR surveillance, highlighted the potential of LAMP for rapid and accurate detection of resistant pathogens, especially in resource-limited settings [15]. In 2019, WHO’s Global Antimicrobial Resistance Surveillance System (GLASS) continued to endorse LAMP-based methods, noting their applicability across various laboratory capacities for enhancing AMR surveillance and molecular diagnostics [16]. Antimicrobial resistance (AMR) describes the phenomenon whereby microorganisms evolve to withstand the inhibitory or lethal effects of antimicrobial agents that were once effective against them [17]. Therefore, developing a rapid, sensitive, specific, and cost-effective method using resistance gene markers is critical for effective AMR surveillance. Implementing a One Health approach, which integrates human, animal, and environmental health perspectives, is essential for comprehensive AMR control strategies [18]. One Health embodies a unified, interdisciplinary framework that emphasizes the pivotal links among human, animal, and environmental health domains [19]. This approach facilitates coordinated surveillance and control measures, resource optimization, and stronger partnerships across sectors.

Real-time loop-mediated isothermal amplification (RT-LAMP) is a sensitive molecular technique that merges turbidimetry with isothermal nucleic acid amplification to identify DNA/RNA targets at a constant temperature. The ‘real-time’ designation reflects its ability to immediately detect amplification products through turbidity (cloudiness) or visual colorimetric shifts or fluorescence signals, making it highly suitable for field-based or point-of-care testing [20]. In recent years, several RT-LAMP assays have been developed to detect AMR genes, such as *vanA*, *blaOXA-23*, and *msrA*, primarily in clinical isolates or pure cultures [21,22,23]. However, most of these studies focused on a narrow range of resistance genes and lacked validation in complex biological matrices. In contrast, our study presents a novel RT-LAMP assay specifically targeting the tetracycline resistance gene *tet*(M) in *Enterococcus* species, a gene widely distributed across clinical, animal, and environmental sources. Notably, our work extends beyond standard assay development by validating the RT-LAMP assay in spiked human and animal urine samples, demonstrating its practical applicability across One Health sectors. This advancement, along with the use of real-time turbidimetry and multiple endpoint detection modalities, sets our study apart from previously published LAMP-based diagnostic methods.

Therefore, this study aimed to develop an RT-LAMP assay for the detection of the tetracycline resistance gene *tet*(M) and to assess its performance using human and animal samples spiked with *tet*(M)-positive *Enterococcus* species.

## 2. Materials and Methods

### 2.1. Bacterial Strains

A total of 52 tetracycline resistance gene *tet*(M)-positive *Enterococcus* species isolates were used in this study. The *Enterococcus* species harbouring the *tet*(M) gene comprised 34 *E. faecalis* (including *E. faecalis* ATCC 29212), 12 *E. faecium*, 5 *E. gallinarum,* and 1 *E. casseliflavus*. For the specificity analysis, two *tet*(L) genes-positive *E. faecalis* and *E. gallinarum* (clinical isolates) were incorporated with non-enterococci bacterial isolates. The non-enterococci isolates included *Staphylococcus aureus* ATCC25923, *Klebsiella pneumoniae* ATCC 700603, *Streptococcus pyogenes* ATCC 19615, *Streptococcus agalactiae* NCTC8017, *Escherichia coli* ATCC 25922, *Pseudomonas aeruginosa* ATCC 27853, and *Klebsiella pneumoniae* ATCC1705. These reference strains were purchased from the Public Health England (PHE) culture collections (National Collection of Type Cultures [NCTC]) and American Type Culture Collection (ATCC). A total of 11 *tet*(M)-positive enterococci strains were used for the performance evaluation of the RT-LAMP assay using human and animal urine samples spiked with those isolates.

### 2.2. DNA Extraction for RT-LAMP Development

Genomic DNA for the LAMP assay was extracted using the water boiling method. Briefly, a single colony of bacteria was collected from the sheep blood agar (HiMedia Laboratories Pvt. Ltd., Mumbai, India) after 24 h of incubation and placed into an Eppendorf tube (EP tube) containing 0.2 mL of double distilled water (ddH_2_O). The EP tube was then heated in a boiling water bath for 8–10 min and centrifuged at 12,000× *g* for 4 min. The resulting supernatant containing the genomic DNA served as the template for the LAMP assay development.

### 2.3. Designation of LAMP Primers

**Figure 1:**
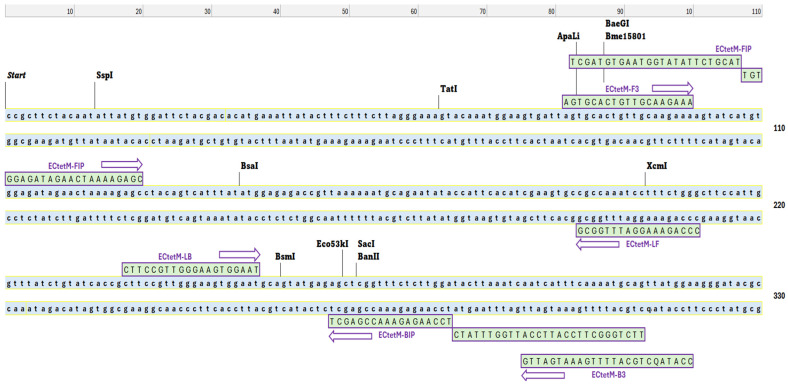
Designation of LAMP primers. The loci of the nucleotide sequences (purple arrows) were used for the loop-mediated isothermal amplification (LAMP) primers. These primers targeted the tetracycline resistance *tet*(M) gene in enterococci. F3 and B3 were used as the outer primers, while FIB and BIP were chosen as the inner primers. LB and LF (loop primers) were used to amplify the reaction.

The conserved nucleotide sequence of the selected enterococci DNA was obtained from the National Centre for Biotechnology Information (NCBI), while oligonucleotide primers for the LAMP assay were designed using the PrimerExplorer v.5 software (http://primerexplorer.eiken.co.jp/lampv5e/index.html, accessed on 20 July 2023). The primer sets were highly specific and targeted six *tet*(M) gene regions during the LAMP reaction. They consisted of two outer primers (F3 and B3) and two inner primers (FIP and BIP), respectively. The forward inner primer (FIP) consisted of the F2 sequence linked to the reverse complement of the F1c sequence, while the BIP contained the B2 sequence linked to the reverse complement of the B1c sequence (Table 1 and Figure 1). To further enhance the amplification process and improve the efficiency of the *tet*(M) RT-LAMP assay, loop forward (LF) and loop backward (LB) primers were included. Following the established protocols, extracted bacterial DNA, the master mix, forward primers, reverse primers, and Millipore water were introduced into the PCR machine using EP tubes after setting the appropriate PCR conditions.

### 2.4. Multiplex PCR for the Detection of Tetracycline Resistance Genes in Enterococci

A total of 105 tetracycline-resistant enterococci were screened for tetracycline resistance *tet*(M) and *tet*(L) genes by the Kirby–Baur method and epsilometer test (E-test). Multiplex PCR was used with respective primers (Table 2). The DNA was extracted using the boiling method [24], and its amount was quantified at A260 by UV spectrophotometry. Briefly, the PCR mixture consisting of 1 µg template DNA, 1× PCR buffer, 2.5 U *Taq* DNA polymerase, 300 μL each of the deoxynucleotides, and ddH_2_O was also prepared. The *tet*(L) and *tet*(M) primer concentrations were optimized both at 1 µL. Amplification was performed in an MJ thermocycler model PTC200 (Fisher Scientific, Ottawa, ON, Canada) with an initial denaturation at 94 °C for 5 min, followed by 35 cycles of 94 °C for 1 min, 55 °C for 1 min and 72 °C for 15 min. The PCR products were sequenced (Apical Scientific Sdn Bhd, Malaysia) and analysed using the Basic Local Alignment Search Tool (BLAST) for the *tet* genes.

### 2.5. Optimization of LAMP for the tet(M) Resistance Gene in Different Reaction Conditions

The LAMP assay was optimized at different concentrations of primers and other reagents using a Nebiocalculator (https://nebiocalculator.neb.com, accessed on 15 November 2023). The LAMP assay was finally performed in 25 μL of the reaction mixture containing 12.5 μL of the 2X Reaction Mix [40 mM Tris-HCl, 20 mM KCl, 20 mM (NH_4_)_2_SO_4_, 16 mM MgSO_4_, 0.2 wt% Tween 20, 1.6 M betaine, and 2.8 dNTPs], 0.32 μL (8U) of *Bacillus stearothermophilus* (*Bst*) DNA polymerase, and 1 μL of fluorescent dye (calcein) and MnCl_2_ (Eiken Co. Ltd., Japan). Moreover, the best optimization of the primers was chosen as follows: 0.2 μL of F3 and B3 primers (5 pmol for each), 1.6 mM of FIP and BIP primers, and 0.8 mM of LB and LF primers. Finally, 2 μL of the target DNA template and 1 μL ddH_2_O were added to the LAMP reaction assay.

Each LAMP reaction was optimized at different amplification times between 10 and 60 min and temperatures between 60 and 65 °C, respectively. All the reactions were performed in an LA-500 Turbidimeter Machine (Eiken Chemical Co., Ltd., Tokyo, Japan). The turbidity of each LAMP tube reaction was subsequently measured at 650 nm, and the signals were then recorded and generated in the form of sigmoidal graphs. The LAMP assay products were examined using various modalities, including color change (orange to green), naked-eye observation of turbidity, and green fluorescence under UV light. Lastly, 1.5% agarose gel electrophoresis was used to analyse the LAMP products [6].

### 2.6. Specificity and Sensitivity of the tet(M) RT-LAMP Method

We evaluated the specificity of the LAMP assay using 11 bacterial isolates (Table 3). These isolates were tetracycline-sensitive by the Kirby-Bauer method, except for *E. faecalis* ATCC 29212 and *E. faecalis* clinical isolates. Additionally, two *tet*(L)-positive and *tet*(M)-negative *Enterococcus* species, namely *E. faecalis* (Accession no. EF50) and *E. gallinarum* (Accession no. EG90), were also included in the study. The *tet*(L) gene was included in the study to evaluate the specificity of the *tet*(M) RT-LAMP assay. By incorporating *tet*(L)-positive but *tet*(M)-negative *Enterococcus* strains, we would be able to confirm that the assay specifically amplifies only the *tet*(M) gene without cross-reactivity.

The sensitivity of the *tet*(M) gene LAMP assay was evaluated using 10-fold serial dilutions ranging from 10^2^ to 10^−6^ ng/µL of the total genomic DNA of *E. faecalis* isolate ATCC 29212. A micro-spectrophotometer (Thermo Fisher Scientific Inc., Wilmington, DE, USA) was used to measure the concentration of genomic DNA. We added distilled water until the final concentration reached 100 ng/mL. The amplification process was performed in accordance with the optimized LAMP assay. A similar protocol was used for the conventional PCR assay, utilising only the F3 and B3 primers to compare the sensitivity of *tet*(M) RT-LAMP assay.

### 2.7. LAMP Validation on tet(M)-Positive Enterococcus Species

The RT-LAMP assay was used to validate the presence of the *tet*(M) gene in 52 *tet*(M)-positive *Enterococcus* species. *E. faecalis* ATCC 29212 was used as a positive control, while ddH_2_O was the negative control. The LAMP products were purified and sent to the Apical Company (Apical Scientific Sdn. Bhd, Klang, Selangor, Malaysia) for sequencing and analysis for the similarity index.

### 2.8. Evaluation of the tet(M) RT-LAMP Assay in Spiked Human and Animal Urine Samples

Before the procedure, urine specimens were taken from a human volunteer with the defined inclusion and exclusion criteria. For instance, a healthy individual with no underlying medical conditions and antibiotic-free for at least 2 weeks before urine sampling. Individuals with a history of catheterization in the past few weeks were excluded. For animal specimens, clean-catch urine sampling from a healthy cow was performed by the well-experienced staff of the Department of Farm and Exotic Animal Medicine and Surgery Specialist, Faculty of Veterinary Medicine, UPM, Serdang, Selangor, Malaysia. The validation process was performed in accordance with established protocols by Aglietti, et al. [25].

Urine samples were freshly obtained, brought to the laboratory, and kept at 2–4 °C for further processing. The sterility of urine samples was confirmed before the spiking procedure by the absence of bacterial growth on selective media. The urine samples of humans and cattle were spiked accordingly with the enterococci, as described by Bowen and Volchok [26]. Briefly, 50 µL of 1 × 10^8^ CFU/mL of bacteria were added to 4950 µL of urine sample. Each test tube received an aliquot of 5 mL urine sample containing 1 × 10^5^ CFU/mL *tet*(M)-resistant *Enterococcus* species. A different type of *tet*(M) gene-positive *Enterococcus* species was added in each test tube. There were 4 *E. faecalis*, 3 *E. faecium*, 3 *E. gallinarum*, and 1 *E. casseliflavus*. The procedure was repeated in triplicate. The DNA extraction of the isolates was performed using the boiling procedure, and the isolates were then processed with the *tet*(M) RT-LAMP assay, as described above.

## 3. Results

### 3.1. Optimization of tet(M) RT-LAMP Assay at Different Temperatures

**Figure 2:**
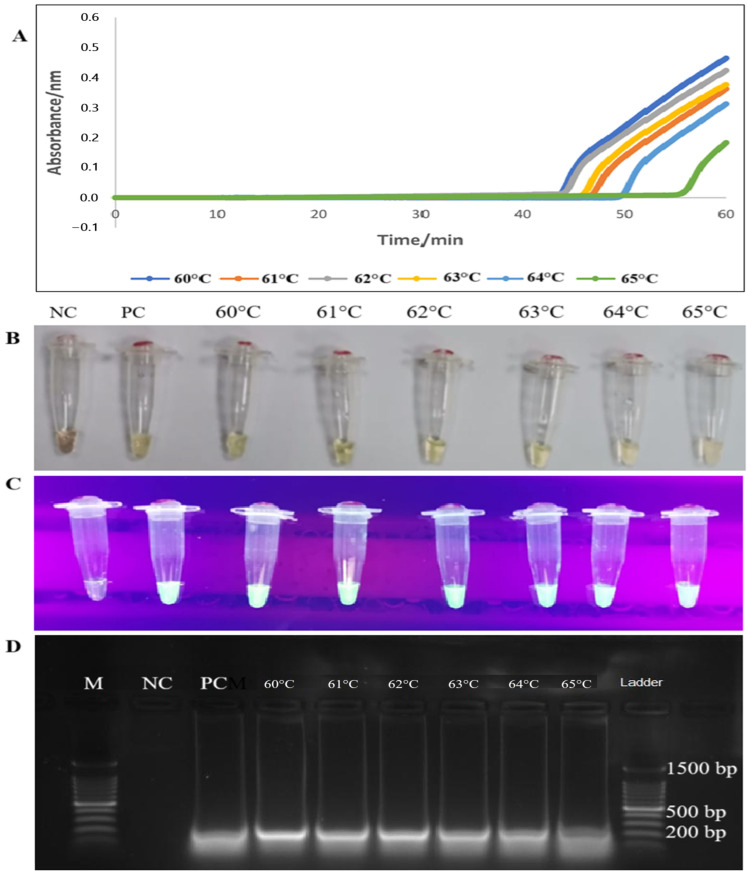
Optimization of *tet*(M) RT-LAMP assay according to temperatures using four techniques for detecting RT-LAMP assay products (**A**–**D**). (**A**) Lines with different colours represented the generation of PCR products at different temperature readings at 60 °C, 61 °C, 62 °C, 63 °C, 64 °C, and 65 °C. (**B**,**C**) Colour changes from orange to green and green fluorescence (calcein dye) with UV light were observed in the reaction tubes. (**D**) The LAMP PCR products of 200 bp were observed on 1.5% gel electrophoresis. M: a 100 bp ladder (Eco Plus, Selangor, Malaysia), PC: positive control (*E. faecalis* ATCC 29212), NC: negative control (distilled water).

Following the optimization of the *tet*(M) gene primer set, the temperature was determined for the best performance of *Bst* DNA polymerase, which ranged from 60 °C to 65 °C. All six temperatures (60 °C, 61 °C, 62 °C, 63 °C, 64 °C, and 65 °C) showed an apparent increase in amplification between 43 to 60 min based on sigmoidal curves. In the present study, the best temperature of the LAMP assay for detecting *tet*(M) was found to be 60 °C by forming a sigmoidal graph with an absorbance maximum of 0.5 nm within 60 min based on established procedure [27]. Sigmoidal graph formation was also supported by the visual observation of the LAMP reaction products in the reaction tubes by orange to green color change, green fluorescence under a UV lamp, and 200 bp PCR products on 1.5% gel electrophoresis (Figure 2).

### 3.2. Optimization of tet(M) RT-LAMP Assay at Different Reaction Times

**Figure 3:**
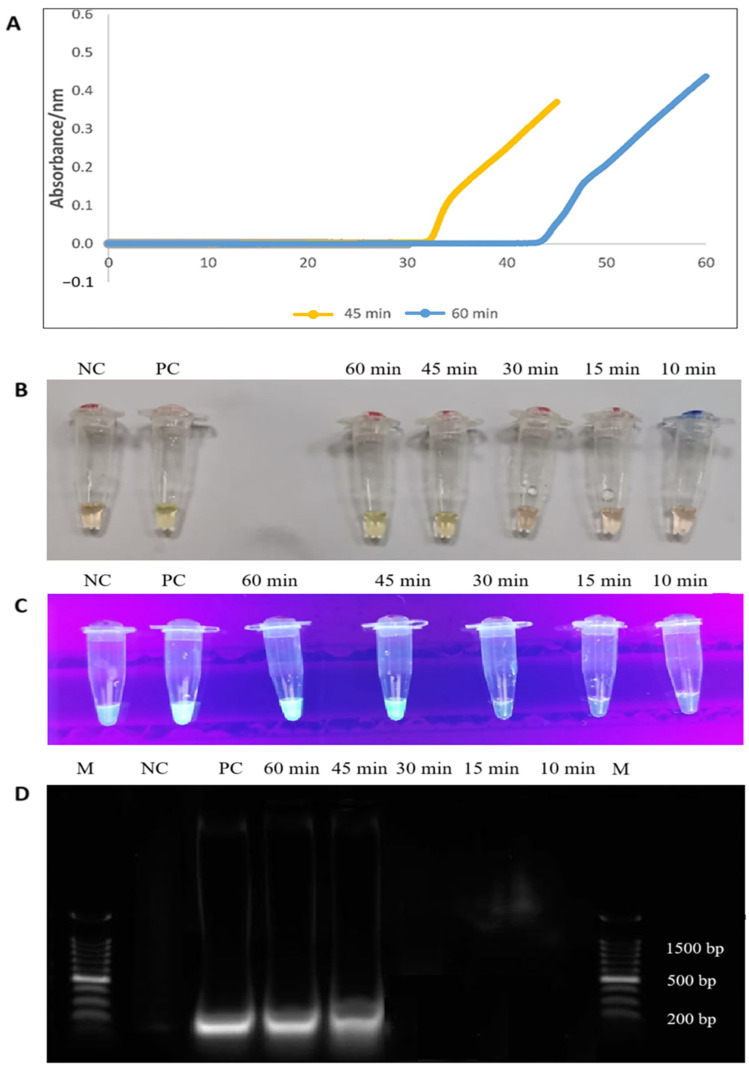
(**A**) Complete formation of sigmoidal graphs within 45 and 60 min (orange and blue line colours). (**B**,**C**) The colour changes from orange to green, and the formation of green fluorescence was observed by the naked eye and under UV light, respectively. (**D**) A 200 bp of PCR products were observed on 1.5% gel electrophoresis. M: a 100 bp ladder (Eco Plus, Selangor, Malaysia), PC: positive control (*E. faecalis* ATCC 29212), NC: negative control (distilled water).

The RT-LAMP experiment was conducted at various intervals to determine the most appropriate duration. The time points were set at 10, 15, 30, 45, and 60 min and monitored using an LA-500 turbidimeter (Eiken Chemical Co. Ltd., Tokyo, Japan). Figure 3 depicts the formation of two sigmoidal graphs as early as 33 and 43 min, and the complete amplification of PCR products was achieved at the 45 and 60-min time points, respectively. Considering the maximum OD reading, the second sigmoidal graph obtained within 60 min was selected. The generation of PCR products was also observed by an orange-to-green colour change (visually) and green fluorescence in the reaction tubes under UV light within 45 min of RT-LAMP assay. Finally, the 200-bp PCR products were observed on 1.5% gel electrophoresis (Figure 3).

### 3.3. Determination of the Specificity and Sensitivity of RT-LAMP Compared to Conventional PCR Method

#### 3.3.1. Specificity Testing of RT-LAMP

**Figure 4:**
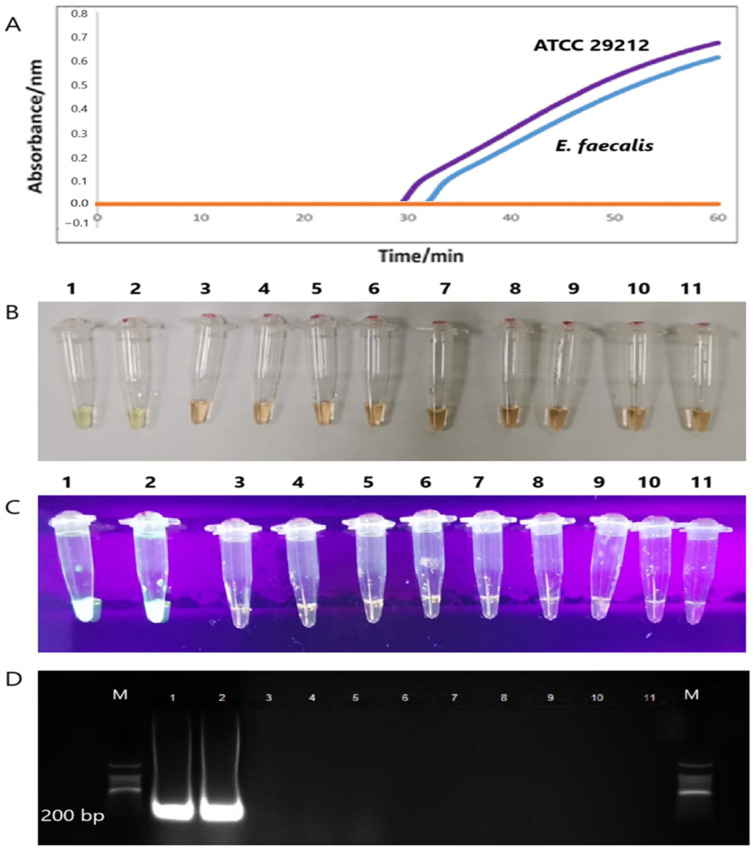
(**A**) Two sigmoidal graphs generated by the amplification of the *tet*(M) gene of the *E. faecalis* ATCC 29212 (purple line) and *E. faecalis* clinical (blue line). (**B**,**C**) The color changes from orange to green, and the formation of green fluorescence was observed by the naked eye and under UV light, respectively. (**D**) Only two 200 bp PCR bands were observed in Lanes 1 and 2. M: a 100 bp ladder (Eco Plus, Selangor, Malaysia), Lane 1: *E. faecalis* ATCC 29212, Lane 2: *E. faecalis* clinical isolate), Lane 3: *K. pneumoniae* ATCC 1705, Lane 4: *S*. *pyogenes* ATCC 19615, Lane 5: *S*. *agalactiae* NCTC 8017, Lane 6: *E. coli* ATCC 25922, Lane 7: *P*. *aeruginosa* ATCC 27853, Lane 8: *K. pneumoniae* ATCC 700603, Lane 9: *S. aureus* ATCC 25923, Lane 10: *E. gallinarum* clinical isolate, and Lane 11: *E. faecalis* clinical isolate.

The specificity of the *tet*(M) gene RT-LAMP assay revealed the formation of sigmoidal graphs for the DNA extracts of *E. faecalis* (ATCC 29212) and *E. faecalis* clinical isolate only. DNA extracts from other bacterial strains did not yield sigmoidal graphs. Interestingly, no sigmoidal graphs were observed for the two *tet*(L)-positive *E. faecalis* and *E. gallinarum*, which reflects the high specificity of the *tet*(M) RT-LAMP assay in the present study. The findings were also validated by visual observation of orange to green, green fluorescence under the UV light, and 200-bp PCR bands observed on 1.5% gel electrophoresis (Figure 4).

#### 3.3.2. Sensitivity Testing

**Figure 5:**
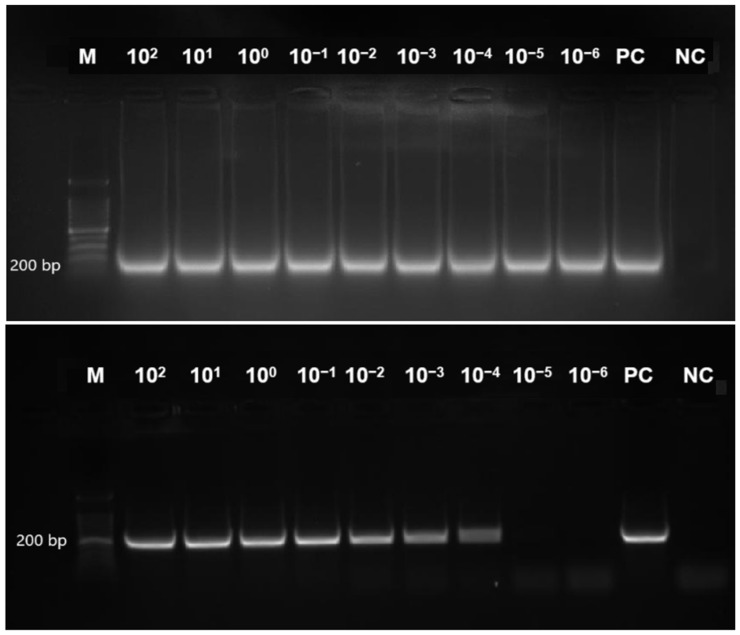
This figure presents a comparative analysis of the sensitivity of RT-LAMP and conventional PCR assays for detecting the tet(M) gene in Enterococcus faecalis ATCC 29212. A 200 bp PCR product was observed on 1.5% gel electrophoresis. Lane M contains a 100 bp ladder (Eco Plus, Selangor, Ma-laysia); Lanes 1–9 represent decreasing DNA concentrations (10^2^ to 10^−6^ ng/µL); PC denotes the positive control (E. faecalis ATCC 29212, undiluted), and NC indicates the negative control (distilled water).

The *tet*(M) gene detection sensitivity by the RT-LAMP assay was performed using the genomic DNA of *E. faecalis* (ATCC 29212). Sigmoidal graphs were observed in all the reaction tubes with 10-fold serial dilutions of DNA from 10^2^, 10^1^, 10^0^, 10^−1^, 10^−2^, 10^−3^, 10^−4^, 10^−5^, and 10^−6^ ng/ μL (Supplementary Figure S1). The findings were complemented by the two different visual modalities, namely, naked-eye observation of orange-to-green colour change and green fluorescence under the UV light in all reaction tubes. In comparison, the *tet*(M) RT-LAMP assay detection limit was lower than that of the conventional PCR assay (0.001 pg/ μL versus 0.1 pg/ μL). Figure 5 compares the detection limits of *tet*(M) RT-LAMP and conventional PCR assay on a 1.5% gel electrophoresis.

### 3.4. Validation of the tet(M) RT-LAMP Assay with Human and Animal Urine Samples Spiked with Enterococcus Species

**Figure 6:**
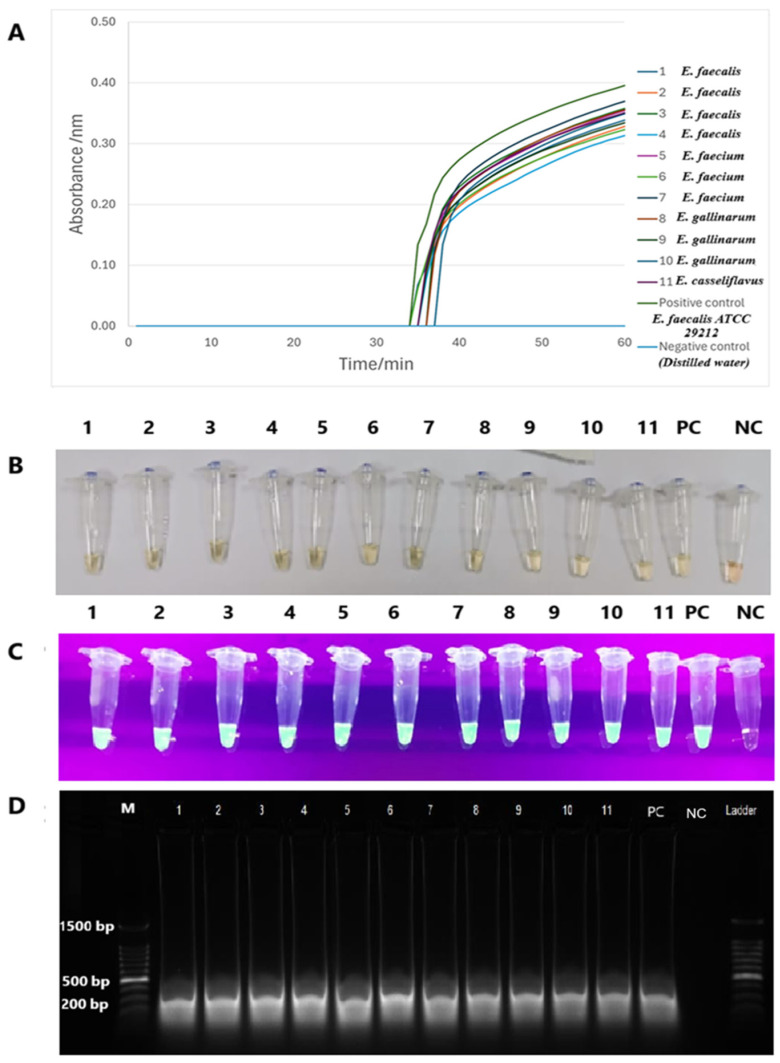
The validation performance of *tet*(M) RT-LAMP assay in spiked human urine. (**A**) The formation of sigmoidal graphs with different colours was observed from all reaction tubes. (**B**,**C**) Visual observations by the colour change from orange to green and green fluorescence through the naked eye and under the UV light, respectively. (**D**) A 1.5% gel electrophoresis revealed 200 bp PCR bands. M: a 100 bp ladder (Eco Plus, Selangor, Malaysia). Lane M: a 100 bp ladder (Eco Plus, Selangor, Malaysia), Lane 1: *E. faecalis*, Lane 2: *E. faecalis*, Lane 3: *E. faecalis*, Lane 4: *E. faecalis*, Lane 5: *E. faecium*, Lane 6: *E. faecium*, Lane 7: *E. faecium*, Lane 8: *E. gallinarum*, Lane 9: *E. gallinarum*, Lane 10: *E. gallinarum*, and Lane 11: *E. casseliflavus* PC: positive control (*E. faecalis* ATCC 29212), NC: negative control (distilled water).

**Figure 7:**
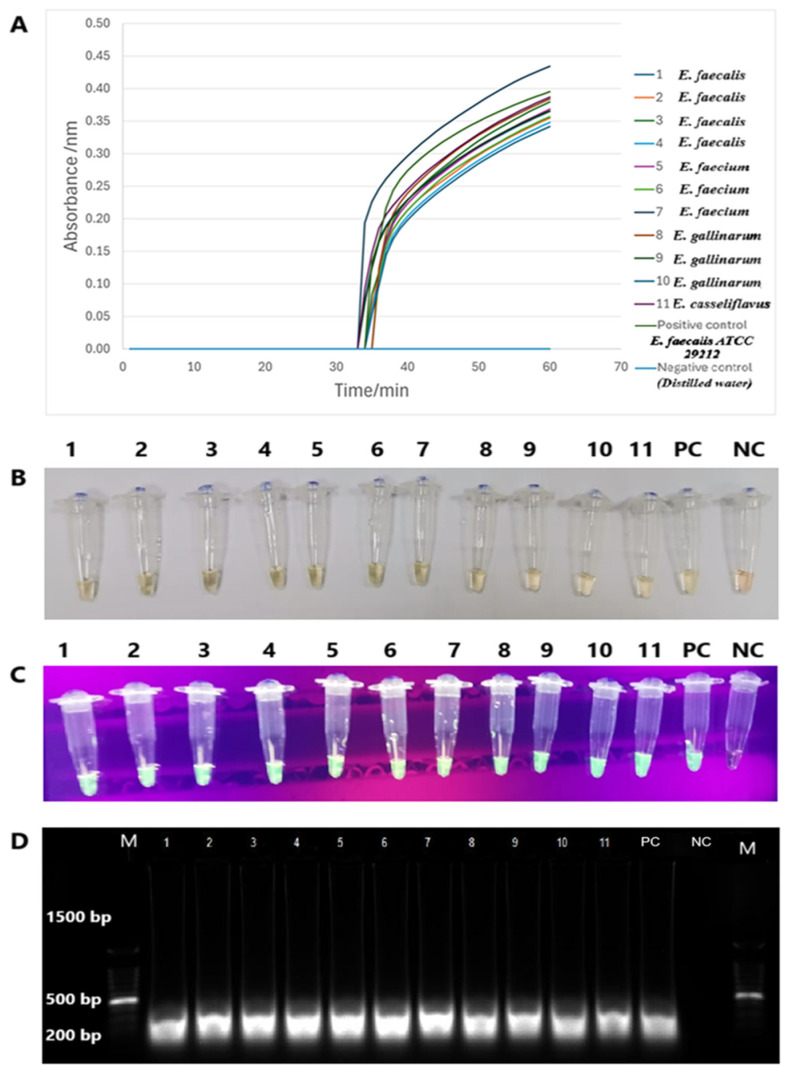
The validation performance of *tet*(M) RT-LAMP assay in spiked animal urine. (**A**) Sigmoidal graphs with different colours were generated from all reaction tubes. (**B**,**C**) The presence of colour changes from green to yellow and green fluorescence was observed by the naked eye and under UV light, respectively. (**D**) PCR products of 200 bp were observed on a 1.5% gel electrophoresis. M: a 100 bp ladder (Eco Plus, Selangor, Malaysia), Lane M: a 100 bp ladder (Eco Plus, Selangor, Malaysia), Lane 1: *E. faecalis*, Lane 2: *E. faecalis*, Lane 3: *E. faecalis*, Lane 4: *E. faecalis*, Lane 5: *E. faecium*, Lane 6: *E. faecium*, Lane 7: *E. faecium*, Lane 8: *E. gallinarum*, Lane 9: *E. gallinarum*, Lane 10: *E. gallinarum*, and Lane 11: *E. casseliflavus* PC: positive control (*E. faecalis* ATCC 29212), NC: negative control (distilled water).

The *tet*(M) RT-LAMP assay performance was validated with 11 strains of *tet*(M)-positive enterococci spiked into human and animal urine samples. Sigmoidal graphs were consistently observed in all *Enterococcus* species and the positive control, *E. faecalis* ATCC 29212. No sigmoidal graph was observed for the negative control. This was further confirmed by visual observation of the orange-to-green color change and green fluorescence under UV light in the reaction tubes. The 200-bp PCR products were also observed on 1.5% gel electrophoresis. Human and animal urine spiked with this *tet*(M)-positive *Enterococcus* species yielded similar findings (Figure 6 and Figure 7).

## 4. Discussion

The implementation of loop-mediated isothermal amplification (LAMP) has enabled the identification of a diverse array of pathogens, including bacteria, viruses, and protozoa, due to its notable rapidity, higher sensitivity, and remarkable specificity [28,29]. In addition to pathogen identification, LAMP has become a crucial molecular technique for detecting AMR, aligning with global AMR initiatives set forth by the World Health Organisation [15,16]. For instance, the LAMP technique has demonstrated efficacy in identifying resistance genes, including *vanA* in vancomycin-resistant *Enterococcus* and carbapenemase genes in carbapenem-resistant *Acinetobacter baumannii* [21,22]. Notably, the adaptability inherent in the LAMP may facilitate its incorporation into resource-limited environments, thereby enhancing timely antimicrobial resistance management through a coordinated approach within the One Health framework.

*Enterococcus* species not only established biological markers for recreational water quality assessment but also reservoirs of antibiotic resistance genes (ARGs) [30,31,32]. These species are promising candidates for the surveillance of antibiotic-resistance genes, including *tet*(M). Furthermore, *tet* genes have been extensively investigated across diverse contexts, revealing that enterococci exhibit significant bio-adaptability and a propensity for the rapid emergence of resistant strains upon exposure to antibacterial agents, particularly tetracyclines [5,33,34,35,36]. In the present study, we established an RT-LAMP assay targeting the *tet*(M) resistance gene in enterococci, serving as a proof of concept for an AMR surveillance tool within the framework of a One Health approach. The *tet*(M) gene is primarily carried by mobile genetic elements and is often disseminated among identical and different bacterial species via horizontal gene transfer [9,37]. The advancement of the *tet*(M) RT-LAMP assay is particularly relevant in light of the necessity for vigilant surveillance of this resistance determinant, which has arisen from the indiscriminate utilisation and escalating demand for tetracyclines in both hospital and aquaculture environments, as well as in animal husbandry for therapeutic and metaphylactic applications [11,38,39,40].

In the RT-LAMP assay, the designation “real-time” signifies that the amplification process can be continuously observed and monitored in real-time through various visual readouts [41]. In our *tet*(M) RT-LAMP assay, the visual readout has been enhanced from the conventional naked-eye assessment of turbidity in the reaction tube to the generation of sigmoidal graphs. This advancement provides a more accurate visual representation of the onset of amplification of the gene of interest. To ensure the reliability of the *tet*(M) RT-LAMP assay, primers targeting six distinct loci of the *tet*(M) gene in enterococci were designed (Figure 1). We employed three distinct sets of primers: the outer primers (F3 and B3), the inner primers (FIP and BIP), and the loop primers (LF and LB), which target multiple binding sites, as detailed in Table 1. The incorporation of loop primers significantly improved the reaction stability, facilitated the detection of additional sequences, and reduced non-specific amplification [23,42]. After establishing the primer sets, we conducted a series of LAMP assays to determine the optimal temperature, evaluating conditions across a range of 60 to 65 °C. We found that the best temperature for our *tet*(M) RT-LAMP assay is 60 °C, even though visual readouts and a PCR product size of 200 bp showed that the *tet*(M) gene was present at all temperatures. The development of a sigmoidal curve at 60 °C exhibited the peak absorbance measurement recorded by the turbidimeter apparatus (Eiken Co. Ltd., Japan). Interestingly, this temperature correlates well with the activity of *B. stearothermophilus* DNA polymerase (*Bst*) in LAMP assays, which critically depends on optimization within the 60 to 65 °C range [27].

Compared to other amplification methodologies, the LAMP assay is characterized by its rapid amplification time. In our study, the *tet*(M) RT-LAMP assay demonstrated the emergence of a sigmoidal graph as early as 33 min, with amplification completion observed at 45 min. The complete development of the sigmoidal curve, characterized by the peak absorbance value indicative of maximum amplification, was achieved within 60 min. The objective is to facilitate the comprehensive amplification of the *tet*(M) gene while minimizing the generation of excessive by-products that may interfere with the detection process [43]. The emergence of sigmoidal graphs at two distinct time points was further corroborated by visual observation of colour change, green fluorescence under UV light, and the presence of a 200 bp PCR product.

Under the optimized conditions, our *tet*(M) RT-LAMP assay demonstrated a high level of specificity in its performance. Sigmoidal graphs were generated exclusively for *E. faecalis* ATCC 29212 and a *tet*(M)-positive *E. faecalis* clinical isolate, while no such graphs were produced for tetracycline-sensitive non-enterococcal species. Notably, two *tet*(L)-positive enterococci did not yield any positive results, further substantiating the high specificity of the RT-LAMP assay for the detection of the *tet*(M) gene exclusively in *tet*(M)-positive enterococci isolates. The implementation of meticulously designed LAMP primers in our assay significantly enhanced the specificity of RT-LAMP for accurately targeting the *tet*(M) gene within the two *tet*(M)-positive isolates. The emergence of a sigmoidal graph corresponds with the precipitation of magnesium phosphate, which occurs subsequent to the amplification of LAMP PCR products. This process can be effectively monitored using a turbidimeter [44]. The visual observation of sigmoidal graphs was corroborated by the observed colour change from orange to green, the presence of green fluorescence under UV light, and the detection of a 200 bp PCR product, as demonstrated by 1.5% gel electrophoresis.

In terms of sensitivity, the *tet*(M) RT-LAMP assay demonstrated a performance that was 100-fold greater than that of conventional PCR methods. Specifically, the *tet*(M) RT-LAMP assay demonstrated a detection limit as low as 0.001 pg/μL, compared to of 0.1 pg/μL for conventional PCR. These findings are consistent with previous studies utilizing LAMP assays to detect resistance genes in bacterial populations. For instance, a LAMP assay employing chromogenic dye for endpoint visual detection demonstrated the capability to identify *tet*(X2/X3/X4/X5) genes in tigecycline-resistant *K. pneumoniae* strains, exhibiting a sensitivity that is 10 to 100 times greater than that of conventional PCR methodologies. The minimum detection limit was established at 0.2 fg/μL, in contrast to the 20 fg/µL threshold observed with conventional PCR [45]. Similarly, in a separate investigation employing a chromogenic dye within the LAMP assay targeting the *msrA* gene in *Staphylococcus aureus* isolates, the sensitivity exhibited was found to be 100 times greater than that of conventional PCR, with thresholds of 100 pg/µL compared to 10 ng/µL [23]. While the sensitivity performances are comparable to those observed in our *tet*(M) RT-LAMP assay, it is advisable to refrain from utilising chromogenic dye alone as the endpoint visual detection method. The limitations of naked-eye turbidity and chromogenic LAMP detection have been well documented, particularly concerning their sensitivity, quantification, and reproducibility, which can result in false outcomes. In contrast, real-time turbidimetry or fluorescence-based detection methods provide enhanced precision, improved sensitivity, and kinetic monitoring capability, thereby facilitating high-accuracy diagnostics [46,47].

In light of establishing the *tet*(M) RT-LAMP assay as a reliable screening method for the rapid identification of the *tet*(M) gene in both human and animal specimens, we evaluated its efficacy for the direct detection of the *tet*(M) gene from urine samples spiked with *tet*(M)-positive *Enterococcus* isolates. The *tet*(M) RT-LAMP assay exhibited exceptional efficacy, successfully identifying the *tet*(M) gene through the generation of sigmoidal curves in all urine samples within a timeframe of less than one hour, achieving a detection rate of 100%. Furthermore, the amplification process initiates at approximately 33 min. The presented graphs are consistent with additional endpoint detection methodologies, such as colorimetric changes, green fluorescence, and identifying a 200 bp amplicon size on a 1.5% gel electrophoresis. Collectively, the results of the present study show that our *tet*(M) RT-LAMP assay is accurate, sensitive, specific, and reproducible.

The current investigation underscores the potential application of the *tet*(M) RT-LAMP assay as a surveillance tool for AMR in human and animal contexts. However, it is imperative to recognize several limitations inherent to the present study. The limited quantity of *tet*(M)-positive enterococci, comprising 52 isolates, alongside the utilisation of spiked urine samples, may constrain the extrapolation of our findings to a broader spectrum of clinical (both human and animal) and environmental specimens. While the precise duration for amplifying the *tet*(M) gene can be quantified by generating sigmoidal graphs, implementing our *tet*(M) RT-LAMP assay necessitates using a turbidimeter, potentially constraining its applicability in fieldwork studies. Nonetheless, the application of colorimetric change utilising calcein dye and green fluorescence can be leveraged in the current investigation. In addition, the utilisation of artificial intelligence (AI)-enhanced colorimetric and UV detection systems presents a promising alternative approach. The integration of AI technologies could substantially improve automation, objectivity, and real-time data processing, effectively mitigating the practical constraints associated with traditional turbidometric LAMP monitoring. Furthermore, subsequent investigations should enhance AI-driven mobile applications and portable diagnostic instruments to advance the accessibility and reliability of LAMP assays across various field environments. Consequently, it is imperative that effective surveillance tools for AMR monitoring are optimised and standardised in future endeavours.

Another limitation of this study is using artificially spiked human and animal urine samples for assay validation, rather than naturally infected clinical or environmental specimens. This approach was necessitated by the limited availability and ethical constraints related to accessing naturally occurring *tet*(M)-positive samples across diverse sources. While the spiking method allowed controlled evaluation of the assay’s performance, it may not fully capture the complexity and variability of real-world samples. Therefore, future studies should aim to validate the assay using naturally infected clinical and environmental samples to enhance its translational relevance and field applicability within the One Health surveillance framework.

In addition to developing rapid and cost-effective tools for AMR surveillance, it is imperative to explore novel therapeutic strategies that target unique bacterial mechanisms. Ezzeddine and Ghssein (2023) proposed an innovative approach focusing on bacterial metallophores, metal-chelating molecules that are essential for bacterial survival and virulence, as a new class of antibiotic targets [48]. Similarly, Luo S et al. (2025) demonstrated that Microcin C7 (McC), a Trojan horse peptide conjugate, can remodel the antibiotic activity spectrum by enabling targeted bacteria to actively import the conjugated antibiotic into their membranes, thereby delivering a potent bactericidal effect [49]. This aligns with our study’s focus on enhancing AMR surveillance using the *tet*(M) RT-LAMP assay, emphasizing the need for both advanced diagnostics and novel therapeutic strategies within the One Health framework.

## 5. Conclusions

The developed RT-LAMP assay supports the characteristic features of a rapid, sensitive, and highly specific detection technique for the *tet*(M) gene in tetracycline-resistant *Enterococcus* species. Its performance is further enhanced by the generation of sigmoidal graphs that could offer clear, real-time results within 60 min. These features could be used as a screening tool for rapid AMR surveillance, particularly in low-income countries. Despite limitations, the present assay significantly supports the One Health surveillance approach. Nevertheless, large field validations and the integration of portable, AI-assisted diagnostic technologies are warranted in future studies to support global AMR monitoring efforts in alignment with WHO objectives.

## Figures and Tables

**Table 1:** The LAMP primers and their base pair sizes for *tet*(M) RT-LAMP assay.

Primer	Sequence	Size (BP)
F3	5′-AGT-ATC-ATG-TGG-AGA-TAG-AACT-3′	22
B3	5′-CCA-TAA-CTG-CAT-TTT-GAA-ATG-ATTG-3′	25
FIP	5′-GGCACTTCGATGTGAATGGTATAT-TAGCCTACAGTCATTTATATGGAG-3′	48
BIP	5′-GCCAAATCCTTTCTGGGCTTCACC-GAGCTCTCATACTGC-3′	39
LF	5′-CCCAGAAAGGATTTGGCG-3′	18
LB	5′-CTTCCGTTGGGAAGTGGAAT-3′	20

**Table 2:** Tetracycline resistance gene PCR primers.

Gene	Primer Sequence	Base Pair	Reference
*tet*(L)	FW 5′-TCG TTA GCG TGC TGT CAT TC-3′ RV 5′-GTA TCC CAC CAA TGT AGC CG-3′	267	[3]
*tet*(M)	FW 5′-GTTAAATAGTGTTCTTGGAGA-3′ RV 5′-CTAAGATATGGCTCTAACAAA-3′	517	[4]

**Table 3:** List of bacteria used for specificity testing of RT-LAMP assay.

Bacterial Strain	Source
*Enterococcus faecalis*	ATCC 29212
*Enterococcus faecalis*	Clinical isolate
*Staphylococcus. aureus*	ATCC25923
*Klebsiella pneumoniae*	ATCC 700603
*Streptococcus pyogenes*	ATCC 19615
*Streptococcus agalactiae*	NCTC8017
*Escherichia coli*	ATCC 25922
*Pseudomonas aeruginosa*	ATCC 27853
*Klebsiella pneumoniae*	ATCC1705
*Enterococcus faecalis*	Clinical isolate (EF50)
*Enterococcus gallinarum*	Clinical isolate (EG90)

## Data Availability

Data will be available upon reasonable request from the corresponding author.

## Supplementary Materials

The following supporting information can be downloaded at https://www.mdpi.com/article/10.3390/diagnostics15101213/s1, Figure S1: Sensitivity of *tet*(M) RT-LAMP assay performance in four different modalities.
