# Supplementary material for: Development of an RT-LAMP Assay for Detecting tet(M) in Enterococcus Species: Enhancing AMR Surveillance Within the One Health Sectors
Source: Diagnostics (Basel). 2025 May 12;15(10):1213. doi: 10.3390/diagnostics15101213 (PMC12109771; doi:10.3390/diagnostics15101213)
Supplement: Supplementary file 1 [file diagnostics-15-01213-s001.zip › diagnostics-3584120-supplementary.pdf]

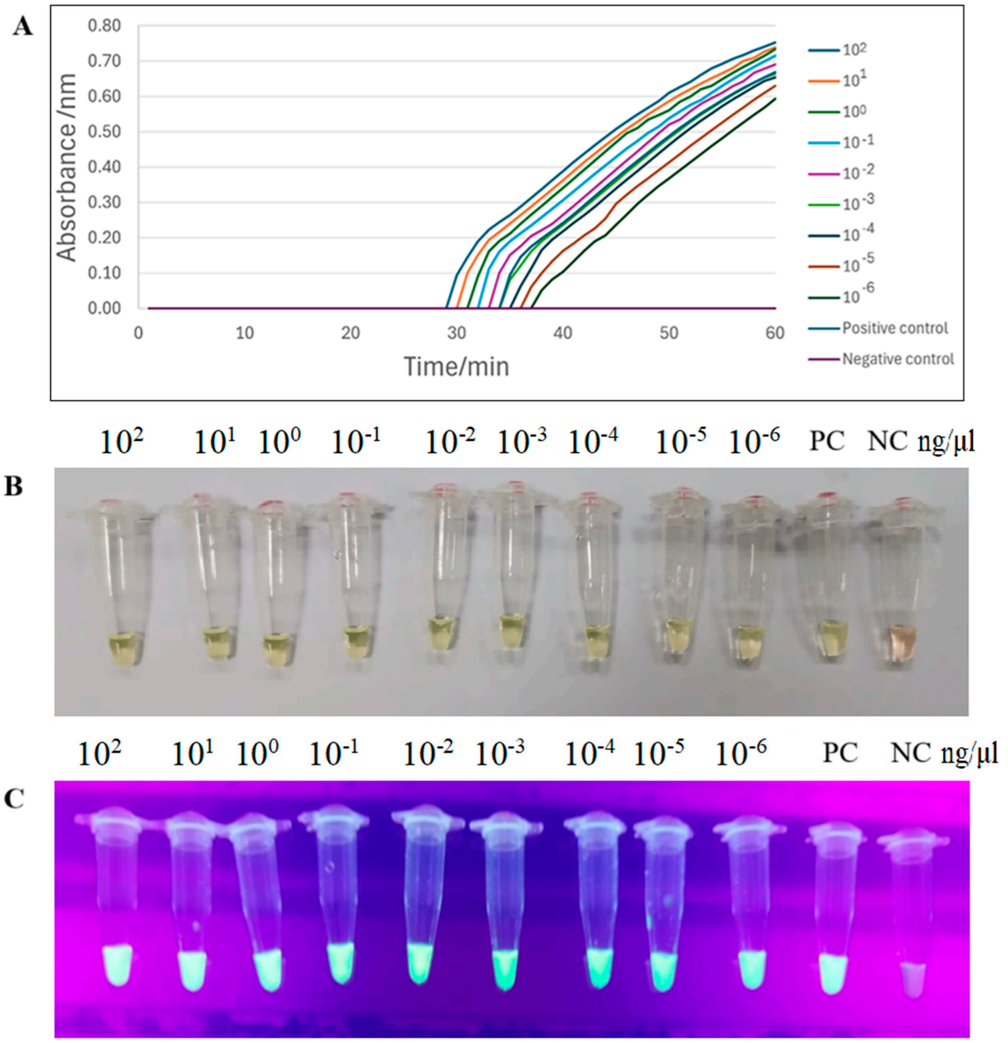

Supplementary Figure 1: Sensitivity of *tet*(M) RT-LAMP assay performance in four different modalities. (A) Sigmoidal graphs with different line colors in a 10-fold serial dilution. (B) Color change from orange to green in all reaction tubes. (C) Green fluorescence in all reaction tubes under the UV light. M: a 100 bp ladder (Eco Plus, Selangor, Malaysia), PC: positive control *(E. faecalis* ATCC 29212), NC: negative control (distilled water)
